# Evaluating the potential of an amelogenin-derived peptide in tertiary dentin formation

**DOI:** 10.1093/rb/rbab004

**Published:** 2021-03-13

**Authors:** Xiu Peng, Sili Han, Kun Wang, Longjiang Ding, Zhenqi Liu, Linglin Zhang

**Affiliations:** State Key Laboratory of Oral Diseases, Department of Cariology and Endodontics, National Clinical Research Center for Oral Diseases, West China Hospital of Stomatology, Sichuan University, Chengdu, China

**Keywords:** tertiary dentin, amelogenin-derived peptide, pulp capping, dental pulp cells

## Abstract

Several novel biomaterials have been developed for dental pulp capping by inducing tertiary dentin formation. The aim of this study was to evaluate the effect of QP5, an amelogenin-based peptide, on the mineralization of dental pulp cells (DPCs) *in vitro* and *in vivo*. The cell viability of human DPCs (hDPCs) after treatment with QP5 was determined using the Cell Counting Kit-8 (CCK-8). Migration of hDPCs was assessed using scratch assays, and the pro-mineralization effect was determined using alkaline phosphatase (ALP) staining, alizarin red staining and the expression of mineralization-related genes and proteins. The results showed that QP5 had little effect on the cell viability, and significantly enhanced the migration capability of hDPCs. QP5 promoted the formation of mineralized nodules, and upregulated the activity of ALP, the expression of mRNA and proteins of mineralization-related genes. A pulp capping model in rats was generated to investigate the biological effect of QP5. The results of micro-computed tomography and haematoxylin and eosin staining indicated that the formation of tertiary dentin in QP5-capping groups was more prominent than that in the negative control group. These results indicated the potential of QP5 as a pulp therapy agent.

## Introduction

Deep caries and trauma can lead to loss of dentin and exposure of dental pulp, which in turn leads to pulpitis and periapical diseases [1]. The dental pulp–dentin complex can induce odontoblasts or odontoblast-like cells to form tertiary dentin under mild external stimulation, thereby preserving the dental pulp from further destruction [2]. The defensive mechanism of dentin–pulp complex for protecting the dental pulp is derived from the biological activity of dental pulp cells (DPCs). In the presence of mild external stimuli, DPCs can differentiate into odontoblasts or odontoblast-like cells and generate dentin-like mineral structures *in vitro* and *in vivo* [3, 4]. The repair of injured pulp involves the migration of endogenous stem cells to the injury site, differentiation of the cells into odontoblast-like cells that can replace necrotic odontoblasts, and secretion of the dentin matrix by the newly differentiated odontoblast-like cells to form tertiary dentin, which protects the pulp from irreversible destruction [3, 5, 6]. However, deep caries or other mechanical disturbances may destroy this line of defense, leading to direct exposure of the pulp and eventually, irreversible pulpitis and pulp necrosis [7, 8]. Therefore, the odontoblastic differentiation of DPCs and stimuli prevention is essential for the formation of reparative dentin [9].

Vital pulp therapy through bioactive and effective measures is recommended for preserving the vitality of damaged pulp tissue in order to maintain their physiological function [10]. Until recently, calcium hydroxide (CH) has been the material of choice for the formation of tertiary dentin [11] because it possesses the ability to release hydroxyl and calcium ions. However, CH has been demonstrated to cause tissue necrosis at the site of direct contact, followed by internal tooth resorption and calcification. These effects may be due to bacterial invasion, formation of tunnel defects in reparative dentin and the high solubility of CH, which leads to loss of the material [12]. Several studies have suggested that the success rates for pulp capping using mineral trioxide aggregate (MTA) are higher than those using CH. However, there are some limitations to using MTA. These include tooth discoloration, prolonged setting time and the formation of a reparative dentin bridge that shows irregular features and structures [5, 13].

New materials including bioactive molecules are being developed for vital pulp therapy [14, 15]. Among these bioactive molecules, several proteins and peptides have been studies based on the requirements of capping materials. For example, bone morphogenetic protein-2 was found to enhance the differentiation of human dental pulp cells (hDPCs) *in vitro* and increase dentin deposition *in vivo* [16, 17]. Dentin matrix protein 1 (DMP1) reportedly guided tissue regeneration in a rat model, wherein dentinal tubules were observed in the new tissue and calcified deposits were observed in the collagenous matrix of this new tissue [18]. An important group of bioactive molecules used in pulp capping comprises enamel matrix proteins and their derivatives. Enamel matrix derivative (EMD), the main components of which are amelogenin, is extracted from the developing enamel tissue. Studies have found that pulp capping with EMD induces markedly greater formation of reparative dentin than that with CH [19, 20]. As the main component of enamel matrix proteins, amelogenin is expressed during all stages of tooth development and is known to inhibit enamel demineralization and promote the remineralization of early enamel caries. In addition, amelogenin can regulate the biological function of DPCs by promoting their differentiation and mineralization, and, thus, help in generating tertiary dentin [21]. However, amelogenin has a high molecular weight and is difficult to extract and purify.

In a previous study, we designed and synthesized several molecules from amelogenin, including a few peptides, which could promote enamel remineralization [22]. It has also been reported that an amino acid present in amelogenin is essential for crystal growth, and that it could promote tertiary dentin formation [19]. We designed QP5, a 22-residue amelogenin-based peptide, which includes five tandem amelogenin (glutamine-proline-X sequence) repeats and a C-terminal ‘tail’ of amelogenin (TKREEVD). The C-terminal sequence of amelogenin has been reported to function in including differentiation of human cementoblast lineage cells [23]. In addition, peptide QP5 is capable of promoting initial remineralization of enamel caries in bovine and rat enamel [24, 25]. According to the pro-mineralization potential of the derived peptide QP5 and the bioregulation effect of C-terminal sequence, we speculated that it might regulate the biological functions of DPCs.

Therefore, the aim of this study was to examined the biological regulation of DPCs by QP5 *in vitro* and *in vivo* and evaluate the potential of QP5 as a new bioactive substance in direct pulp capping treatment.

## Materials and methods

### Ethics statement

All the experimental protocols in this study, including animal experiments were approved by the Ethics Review Committee of West China School of Stomatology and the State Key Laboratory of Oral Diseases (Ethics approval number: WCHSIRB-D-2019-040). All experiments were carried out in accordance with the approved protocol and animal experiments were also conducted in accordance with the guidelines of the Chinese Council on Animal Care.

### Peptide synthesis

The amino-acid sequence of QP5 is QPYQPVQPHQPMQPQTKREEVD. The peptide was compounded by GL Biochem (Shanghai, China) (purity > 95.0%) by standard Fmoc solid-phase chemistry with an Apex 396 peptide synthesizer (AAPPTec, Louisville, KY).

### Cell culture

Human impacted third molars were collected at the West China Hospital of Stomatology. After extraction, the third molars were immediately placed in phosphate-buffered saline (PBS) supplemented with antibiotics. The molars were washed within 15 min of extraction and sectioned to extract the pulp tissue. The extracted pulp tissue was sectioned and digested with 3 mg/mL type I collagenase for 1 h. The mixture was subsequently centrifuged at 1000 rpm for 5 min, and the sediments were collected. We transferred the digested tissue to T-25 flasks containing culture medium with 5% CO_2_, maintained at 37°C. The culture medium contained high-glucose Dulbecco's modified Eagle’s medium (HyClone, Logan, UT), 10% foetal bovine serum (Gibco, Grand Island, NY) and antibiotics (penicillin 100 U/mL and streptomycin 100 μg/mL). We used third passage cells in this study.

The hDPCs were seeded in a growth medium in six-well plates at a density of 1 × 10^4^ cells/cm^2^ for the induction of mineralization. Subsequently, the medium was replaced with mineralization medium (MM) containing 10 nmol dexamethasone (Sigma-Aldrich), 10 mmol sodium β-glycerophosphate (Sigma-Aldrich, St. Louis, MO) and 50 μg/mL ascorbic acid in alpha-modified Eagle’s medium supplemented with 10% foetal bovine serum.

### Cell viability assay

The effect of QP5 on the viability of hDPCs was determined using the Cell Counting Kit-8 (CCK-8, Dojindo, Japan), as described in the instructions. hDPCs were seeded onto 96-well culture plates at a density of 3000 cells per well and exposed to various concentrations of QP5 (10, 20, 50, 100, 150 and 200 μg/mL) for 5 days. Absorbance was monitored with a Thermo Scientific Varioskan Flash (Thermo Scientific, Waltham) at 450 nm.

### Cell migration assay

Cell migration was monitored using the scratch wound assay. Briefly, 2 × 10^4^ cells per well were seeded onto six-well culture plates after 8 h. The cell monolayers were wounded with a 200-μL pipette tip, and washed thrice with PBS to remove cell debris. hDPCs were maintained with and without QP5 for 12 and 24 h, respectively. An inverted microscope (Leica, Wetzlar, Germany) was used to measure wound closure using a collection of digital images. Scratched areas were monitored with the Image-Pro Plus 6.0 software (Media Cybernetics, Bethesda, MD).

### Alkaline phosphatase (ALP) staining and activity analysis

The hDPCs cultured for 3, 5 and 7 days were stained with ALP staining (ALP). hDPCs were washed thrice with PBS, followed by fixation with 4% paraformaldehyde for 20 min, subsequently washed with PBS thrice, and finally stained with the BCIP/NBT ALP Colorimetric Kit according to the manufacturer's instructions (Beyotime Shanghai, China). A semi-quantitative assay was performed with the ALP assay kit in accordance with the manufacturer’s protocol (Sigma, St. Louis, MO). Absorbance was monitored using the microplate reader at 520 nm. BCA Protein Assay Kit was used to quantify the protein content (Beyotime Shanghai, China). The total protein content was used to normalize ALP activity.

### Alizarin red staining

Alizarin red staining was performed as follows. The cells were cultured for 14 days, then rinsed with PBS thrice, and fixed with 4% paraformaldehyde. A 40-mM Alizarin red solution was prepared in distilled water (pH = 5.5). The cells in the 12-well plate were gently stirred for 30 min. They were subsequently rinsed with PBS and allowed to dry. An inverted light microscope was used to observe the orange-red calcium nodules (Olympus, Japan).

### Quantitative real-time polymerase chain reaction

All the RNA was isolated from the cells using TRIzol reagent (Takara Bio, Shiga, Japan) after 7 and 14 days of culture. cDNA was synthesized using the RT Reagent Kit (Takara Bio, Shiga, Japan) in accordance with the manufacturer’s instructions. The expression of genes associated with mineralization, i.e. *ALP*, *OCN* (osteocalcin), *DSPP* (dentin sialophosphoprotein), *OPN* (osteopontin)*, DMP1* (dentin matrix protein 1) and *OSX* (osterix) was analyzed using quantitative real-time polymerase chain reaction (qPCR). The sequences of highly purified primers are outlined in Table 1. qPCR was performed using the SYBR Green PCR kit (Takara Bio, Shiga, Japan) and Bio-Rad’s real-time PCR system (Bio-Rad Laboratories, Hercules, CA). The amplification efficiency of different genes was estimated relative to glyceraldehyde-3-phosphate dehydrogenase (GAPDH), which was the control. The experiment was repeated thrice.

**Table 1.** Primer sequences used in the qPCR

Gene symbol	Forward	Reverse
*DSPP*	TTTGGGCAGTAGCATGGGC	CCATCTTGGGTATTCTCTTGCCT
*DMP1*	CAGGAGCACAGGAAAAGGAG	CTGGTGGTATCTTGGGCACT
*OPN*	ATGATGGCCGAGGTGATAGT	ACCATTCAACTCCTCGCTTT
*OSX*	TCTCCATCTGCCTGACTCCT	AGCGTATGGCTTCTTTGTGC
*OCN*	ATTGTGGCTCACCCTCCATC	CCAGCCTCCAGCACTGTTTA
*ALP*	GACCTCCTCGGAAGACACTC	TGAAGGGCTTCTTGTCTGTG
*GAPDH*	GCTCTCTGCTCCTCCTGTTCG	GCGAACACATCCGGCCTGC

### Western blot analysis

After rinsing twice with ice-cold PBS, the cultured hDPCs were lysed with a cell lysis buffer for 15 min on ice, according to the manufacturer’s instructions (Cell Signaling Technology, Beverly, MA). One sample from each dish was mixed with 100 μL lysis buffer. The cell lysates were scraped, collected and subsequently centrifuged at 14 000 rpm for 10 min. The concentration of total proteins was evaluated using the BCA Protein Assay Kit (Beyotime Shanghai, China). The proteins were separated using 10% sodium dodecyl sulfate-polyacrylamide gels and transferred to a polyvinylidene difluoride transfer membrane (Millipore, Bedford, MA). The membranes were blocked with 5% nonfat milk in Tris-buffered saline for 1 h, followed by overnight incubation at 4°C with specific primary antibodies against DSP (Santa Cruz Biotechnology, Inc., Santa Cruz, CA), OPN (Abcam, Cambridge), DMP1 (Santa Cruz Biotechnology, Inc.), OSX (Abcam) and GAPDH (Bimake, Houston, TX) at 1:1000 dilution. The membranes were incubated after three washes with a secondary antibody-horseradish peroxidase conjugate (Sino-American Biotechnology Co., San Diego, CA) for 1 h, and washed three times after that. Enhanced chemiluminescence was used to visualize the membranes. Relative band intensities were analysed by densitometry using ImageJ software. All experiments were repeated thrice.

### Rat model for pulp capping

Sprague-Dawley rats (7–8 weeks old, weighing 200–250 g) were purchased from Chengdu Dashuo Biotechnology Co., Ltd, (Chengdu, China). Direct pulp capping was performed according to the protocol described previously [26]. Fifteen male rats were administered 10% chloral hydrate (3 ml/kg) intraperitoneally, and their teeth were cleaned and disinfected with 75% ethanol. Subsequently, the dental pulps of the maxillary first molars were mechanically exposed. The cavities in the pulp chambers were exposed to the tip of a sterile, size 08 endodontic file. Bleeding was stopped using sterile cotton pellets. The 50 μg/mL QP5 was used in the experimental group, CH (Dycal, York, PA) was used in the positive control group, and PBS was used in the negative control group. The capping materials were placed over the perforation sites for 4 and 8 weeks, and the cavities were sealed with glass–ionomer cement (GC, Fuji IX, Tokyo, Japan) (Fig. 3A).

### Micro-computed tomography evaluation

The rats were sacrificed on weeks 4 and 8, postoperatively. The maxillae of all groups were dissected, and the teeth were prepared for micro-computed tomography (μCT) scanning (μCT50, Scanco Medical AG, Bruettisellen, Switzerland). The μCT parameters were as follows: 70 kVp, 200 μA, 300-ms exposure time per frame, and 7-μm voxel size. Three-dimensional reconstruction imaging software was used to reconstruct image data. Sagittal images were presented according to mineral density. The ratio of total dentin volume (BV) and total tooth volume (TV) was determined.

### Histological evaluation

The rat maxillae were washed with PBS and fixed with 4% paraformaldehyde overnight, followed by decalcified using 10% ethylenediaminetetraacetic acid/PBS solution for 2 months. Each sample was sectioned sagittally at 5 μm thickness, after serial dehydration and paraffin embedding. The sections in each group were stained with haematoxylin and eosin (H&E).

### hDPCs inflammatory model and enzyme-linked immunosorbent assay

The effect of lipopolysaccharide (LPS) on the viability of hDPCs was determined using CCK-8 (Dojindo, Japan) as mentioned above. The concentrations of *Escherichia coli* (*E. coli*) LPS (SigmaAldrich, St. Louis, MO) were set at 0.5, 1, 5, 10 and 20 μg/mL. Cells were incubated with 1, 5 and 10 μg/mL of *E. coli* LPS in the absence or presence of 10 or 50 μg/mL of QP5 for 12 and 24 h. The levels of interleukin-6 (IL-6) and tumor necrosis factor-α (TNF-α) were determined by means of enzyme-linked immunosorbent assay (ELISA) kits (R&D Systems Inc, Minneapolis, MN). In all cases, a standard curve was constructed from the standards provided by the manufacturer. Cytokine levels were normalized to the protein concentration in lysate.

### Statistical analysis

Each experiment was performed at least thrice. SPSS 18.0 software (SPSS, Chicago, IL) was used for statistical analyses of data. One-way analysis of variance was used to determine statistical significance among the groups. *P < 0.05* was considered as statistically significant. All data were presented as mean ± SD.

## Results

### Enhancement of hDPCs migration ability after QP5 treatment

**Figure 1.**
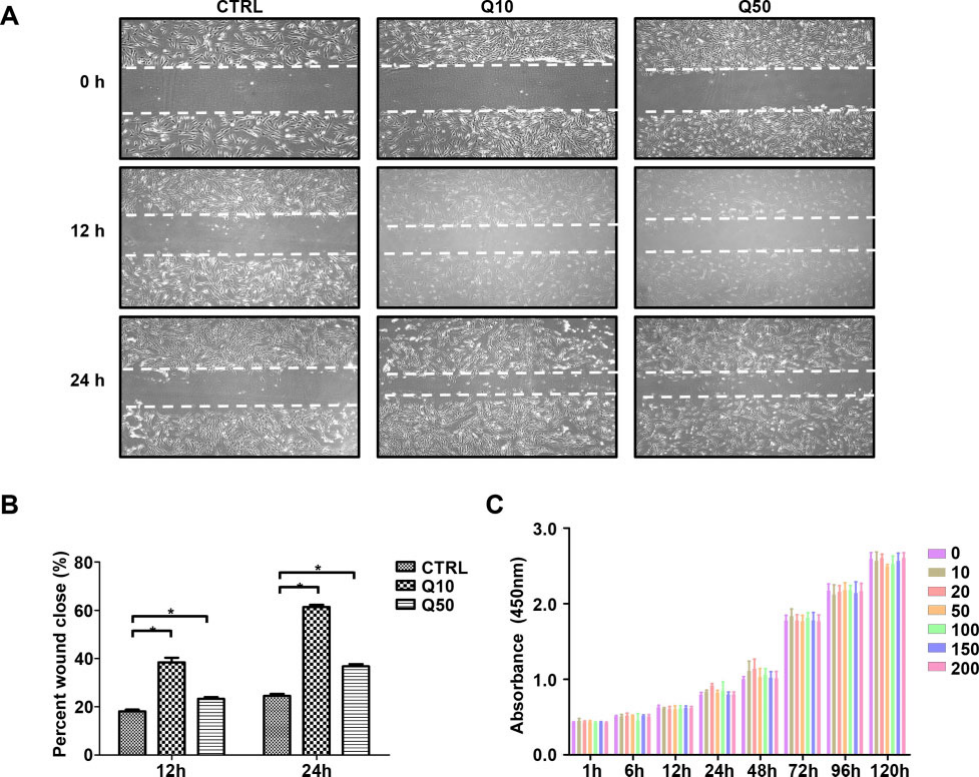
QP5 treatment enhanced the migration ability of hDPCs without cytotoxicity. The effects of QP5 on cytotoxicity and migration of hDPCs. (**A**) Light microscopy images of scratch wound assays, (**B**) Quantitative analysis of migration rates at 12 and 24 h. Scratch wound assays revealed that both 10 and 50 μg/mL concentrations of QP5 significantly promoted the motility of hDPCs (*P < 0.05). (**C**) Effect of QP5 on cell viability determined using the CCK-8 assay, there was no statistically significant difference between the groups (*P* > 0.05). hDPCs, human dental pulp cells; CCK-8, cell counting kit-8.

We used the CCK-8 and scratch wound assays to evaluate cell viability and migration capacity of QP5-treated hDPCs. After incubation with various concentration of QP5 (10, 20, 50, 100, 150, 200 μg/mL), the Cell Counting Kit-8 (CCK-8) assay showed that QP5 did not affect cell viability even at concentration of 200 μg/mL after incubation for 5 days (Fig. 1C). Based on our previous study, 50 μg/mL QP5 promoted the remineralization of enamel caries *in vitro* and *in vivo*. According to the preliminary experimental results, we further performed the scratch wound assay by treating hDPCs with 10 and 50 μg/mL QP5, and found that QP5 could promote the migration of hDPCs (*P < *0.05) (Fig. 1A and B). The concentrations of 10 and 50 μg/mL were used for subsequent experiments.

### Promotion of mineralization of hDPCs after QP5 treatment

**Figure 2.**
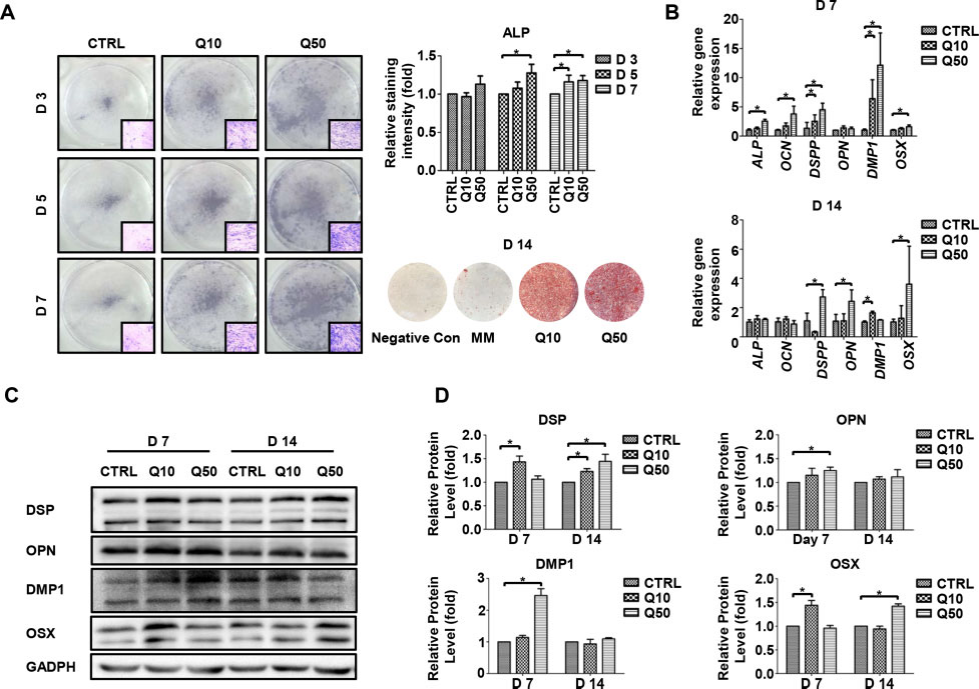
QP5 treatment promoted the mineralization of hDPCs hDPCs were cultured with negative control (Con), 10 μg/mL QP5 (Q10) and 50 μg/mL QP5 (Q50), respectively. (**A**) ALP staining showed promotion of mineralization with Q10 and Q50 of hDPCs on days 3, 5 and 7 after treatment. ALP activity increased in the Q10 and Q50 group on days 3, 5 and 7, with odontogenic induction. On Day 14, alizarin red staining was performed to indicate the effects of QP5 on the mineralization of hDPCs. (**B**) On days 7 and 14 after treatment, the expression levels of genes related to odontogenesis (ALP, OCN, DSPP, OPN, DMP1 and OSX) were evaluated using qPCR. (**C**) The expression levels of the genes related to odontogenesis (DSP, OPN, DMP and OSX) were evaluated using the Western blots on days 7 and 14. (D) The result of the western blot was standardized with GAPDH (*P < 0.05). hDPSs, human dental pulp cells; qPCR, quantitative polymerase chain reaction; ALP, alkaline phosphatase; GAPDH, glyceraldehyde-3-phosphate dehydrogenase.

After hDPCs were cultured with QP5 (10 and 50 μg/mL) in the MM, we first performed ALP staining to detect the ALP activity and alizarin red staining to measure the formation of mineralized nodules. As a result, QP5-treated hDPCs showed stronger ALP staining after 3, 5, and 7 days of mineralization induction, respectively. The quantification of ALP staining intensity revealed that QP5 significantly enhanced hDPC mineralization (*P < *0.05). The number of calcium nodules stained by alizarin red staining in QP5-treated group was increased after culturing hDPCs in QP5-containing MM 14 days, especially at concentration of 50 μg/mL (Fig. 2A).

We further demonstrated the pro-mineralization ability of QP5 by detecting mineralization-related genes both in mRNA and protein levels. The expression of *ALP*, *DSPP*, *OCN*, *OPN*, *DMP1* and *OSX* was detected by real-time quantitative qPCR. The results showed that at the time of 7 days after MM induction, all the mineralization-related genes, except *OPN* were upregulated in the QP5-treated group, especially at the concentration of 50 μg/mL QP5 (*P < 0.05*) (Fig. 2B). Similar gene expression patterns were detected within 14 days of incubation with MM with or without QP5 (Fig. 2B). In addition, proteins related to hDPCs mineralization were detected with the Western Blot after MM culturing 7 and 14 days. As compared with the MM-only group, the protein expressions of DSP, OPN, DMP1 and OSX increased significantly in the QP5-treated group (*P < *0.05) (Fig. 2C and D).

### QP5 induced the formation of hard tissues through direct pulp capping in rats

**Figure 3.**
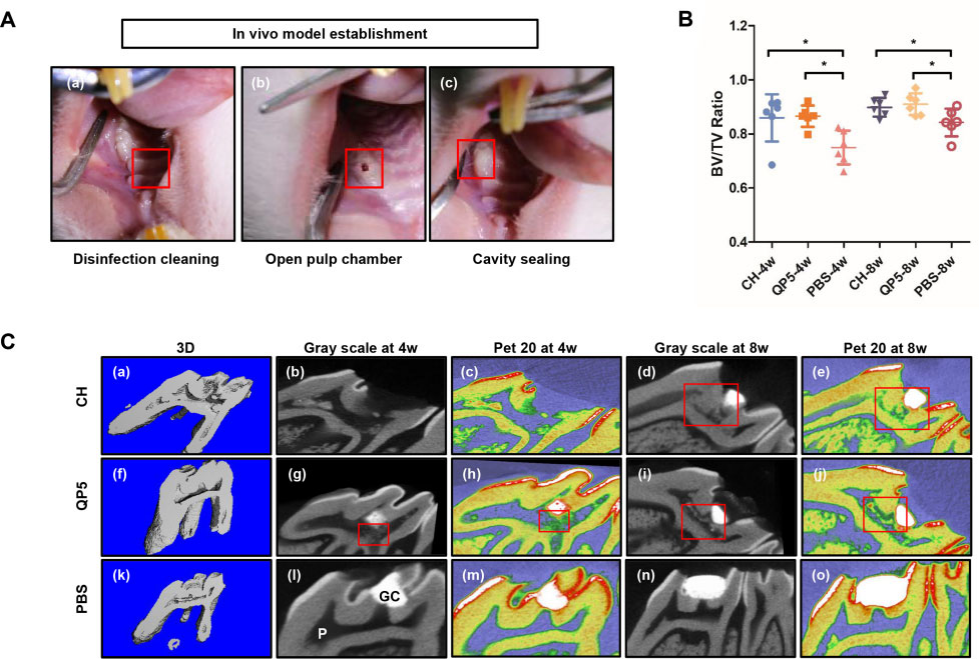
QP5 induced the formation of hard tissues through direct pulp capping in rats. (**A**) Establishment of the pulp capping model. The teeth were cleaned (a) and their pulps were mechanically exposed (b), the cavity was sealed with GIC, after capping with different materials (c). the ratio (BV/TV) of total dentine volume (BV) and total tooth volume (TV) was determined at 4 and 8 weeks (**B**) (*n* = 6, *P < 0.05). (**C**) Representative μCT images of tertiary dentin formation the pulp was exposed mechanically and divided into three groups: the CH (a–e), QP5 (f–j) and PBS control groups (k–o). Three-dimensional sagittal reconstruction images were presented (a, f, k). Representative sagittal μCT images (b, d, g, i, l, n) and color-coded images of tertiary dentin according to the degree of mineral density (c, e, h, j, m, o) are shown. Areas enclosed in the red rectangle indicate tertiary dentin. CH, calcium hydroxide; P, pulp.

We established the direct pulp capping model in rats, to further investigate the pro-mineralization effect of QP5 *in vivo*. The positive control group was treated with CH (the classic capping material), while the negative control group was treated with PBS. The rats were sacrificed for μCT scanning at 4 or 8 weeks after pulp capping. Hard tissue formation was observed and evaluated underneath each capping material. μCT imaging revealed that tertiary dentin formation observed beneath the cavity in the CH and QP5-treated samples, and the effect in 8 weeks was better than that in 4 weeks. However, while little hard tissue formation was observed in the PBS group, either 4 or 8 weeks (Fig. 3C). The CH and QP5-treated group exhibited significantly higher BV/TV ratios compared with the PBS group (*P < *0.05). However, there was no significant difference in the ratio of BV/TV between the CH and QP5-treated groups (*P > *0.05) (Fig. 3B).

### Decreased inflammatory response and obvious tertiary dentin formation in QP5-treated dental pulp

**Figure 4.**
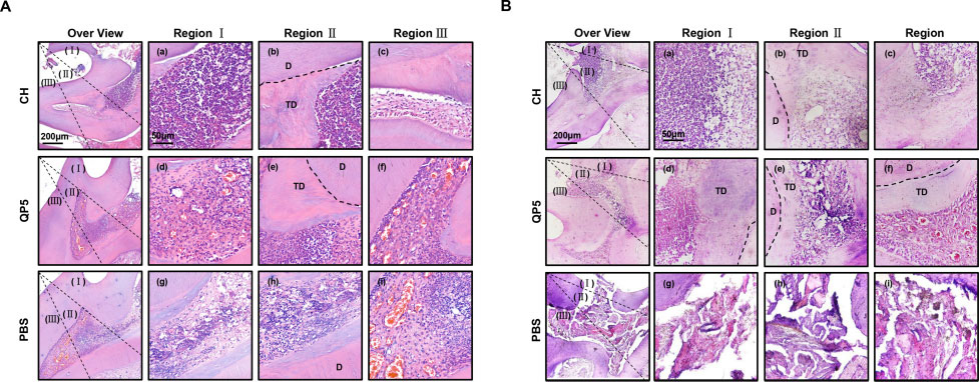
QP5-treated dental pulp showed a decreased inflammatory response and obvious tertiary dentin formation histological images indicating the pulpal response after pulp capping. The rat molars were divided into three groups: the CH, QP5 and PBS groups, based on the different materials used. The pulp was direct capped used different materials after 4 weeks (**A**) and 8 weeks (**B**). Reparative dentin formation was observed adjacent to the existing dentin in the CH and QP5 groups. However, the hard tissue demonstrated an irregular pattern that differed from primary dentin. P, pulp; TD, tertiary dentin; D, dentin; CH, calcium hydroxide; PBS, phosphate-buffered saline.

All the sections were stained with H&E to investigate histological changes in each group. We assessed the results of direct pulp capping by analyzing the structure, tertiary dentin formation and inflammation in each group (Fig. 4). Some pulp necrosis and immune cell infiltration were observed in the CH-treated group, especially directly underneath the penetrated site, while the regular structure of dental pulp could barely be seen (Fig. 4Aa–c). After 8 weeks treated with CH, there was basically no ordered pulp morphology (Fig. 4Ba–c). In the PBS-treated group, part of the dental pulp underwent necrosis, while the other parts still retained the odontoblast layer for 4 weeks (Fig. 4Ag–i). However, the PBS-treated group showed extensive cell necrosis at 8 weeks, and little regular structure of dental pulp was observed (Fig. 4Bg–i). Moreover, pronounced congestion and numerous immune cells had accumulated within the pulp. Direct pulp capping 4 and 8 weeks with QP5 significantly reduced the inflammatory response after dental pulp exposure. Immune cell infiltration showed greater reduction than that in the negative-control group, and no necrosis was observed. Most of the dental pulp tissues maintained regular morphology (Fig. 4Ad–f and 4Bd–f).

The amount and position of the tertiary dentin formation observed on H&E staining were consistent with that of μCT analysis. However, tertiary dentin in the CH-treated group showed greater resemblance to irregular mineralization. Tertiary dentin formation almost did not occur within the necrosed dental pulp in the PBS-treated group. The formation of tertiary dentin was significantly more active in the QP5-treated group, than that in the PBS-treated groups. Tertiary dentin formed in this sample generated a bridge of reparative dentin to block the exposure area and further protect the remaining pulp tissue.

### QP5 had little effect on LPS-stimulated inflammatory model in hDPCs

**Figure 5.**
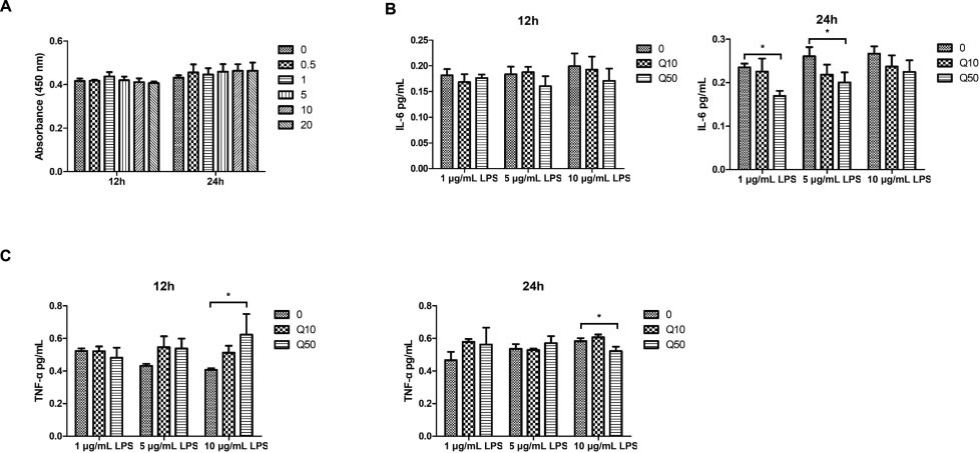
QP5 had little effect on lipopolysaccharide-stimulated inflammatory model in hDPCs LPS-stimulated inflammatory hDPCs were cultured with negative control (Con), 10 μg/mL QP5 (Q10) and 50 μg/mL QP5 (Q50), respectively. (**A**) The CCK-8 assay was used to assess the viability of hDPCs for 12 and 24 h after treatment with different concentrations of *E. coli* LPS. Values are expressed as means ± SD of three replicates of one representative experiment. (**B**) The protein levels of IL-6 were determined with the use of ELISA kits (*P < 0.05). (**C**) The protein levels of TNF-α were determined with the use of ELISA kits. hDPCs, human dental pulp cells; CCK-8, cell counting kit-8; ELISA, enzyme-linked immunosorbent assay.

Lipopolysaccharide was used to establish a pulp inflammation model. The results of CCK-8 showed that 0–20 μg/mL LPS had no obvious effect on hDPCs and 1, 5 and 10 μg/mL LPS was applied to establish a pulp inflammation model (Fig. 5A). ELISA results showed that the expression of IL-6 in DPCs was decreased by 50 μg/mL QP5 at 24 h when LPS concentration was 1 and 5 μg/mL (*P < *0.05). However, when LPS concentration was increased, QP5 did not decrease the expression of IL-6 (*P > *0.05) (Fig. 5B). Although 50 μg/mL QP5 reduced the TNF-α produced by inflammatory hDPCs induced by 10 μg/mL LPS after 24 h, but at 12 h, QP5 had little effect on the expression of TNF-α (*P > *0.05), even 50 μg/mLQP5 increased the expression of TNF-α when LPS was increased to 10 μg/mL (Fig. 5C) (*P < *0.05).

## Discussion

The efficacy of direct pulp capping may be influenced by the biological properties of the materials used and their effects on regeneration of dentin [27]. Biomaterials that mimic the natural repair process are likely to be more effective in the formation of tertiary dentin. At present, small molecular peptides derived from the functional domains of key proteins are designed to simulate the biological effects of large molecular proteins and mimic their function in the process of repair of the injured pulp. The calcium phospholipid-binding protein CPNE7-derived peptide CDP4 could promote osteogenic differentiation of dental pulp stem cells and increase bone regeneration in a mouse calvarial defect model, comparable to full-length CPNE7 [28].

Moreover, Cpne7-DP, a newly developed peptide derived from CPNE7, is able to induce odontoblast-like differentiation *in vitro*, mineralization *ex vivo* and promote tubular dentin formation under both shallow and deep dentinal defects *in vivo*. These researches further illustrated that peptides could be applied as improved alternatives to protein to induce mineralization. Based on the key role of amelogenin in dental biomineralization, we designed *in vitro* and *in vivo* experiments to explore the role of the amelogenin-derived peptide QP5 in the migration and mineralization of DPCs and the formation of tertiary dentin.

Our *in vitro* experiments demonstrate that QP5 significantly promoted cell migration, upregulated the activity of ALP, and induced mineralization of hDPCs, which are important biological functions involved in the repair of damaged dental pulp tissue [29, 30]. According to the sequence of QP5, it is worth noting that a modified C-terminal sequence from amelogenin is included. It has been reported that a formerly identified peptide (STDKTTREEVD, C11), which is also derived from the C-tail of amelogenin, could promote the proliferation of mouse calvarial osteoblastic cells and the osteogenic differentiation of human cementoblast lineage cells by inducing expression of ALP, OCN and BSP [31]. In contrast, peptide derived from amelogenin lacking the C-terminus (rh163) had no significant effect on osteogenic differentiation of human cementoblast lineage cells as compared with untreated controls [23]. In a recent study, we found an antimicrobial peptide TVH-19, which contains a modified C-terminal sequence, could promote odontogenic differentiation of hDPCs *in vitro* and induce tertiary dentin formation in indirect pulp capping [32]. These studies suggest the importance of amelogenin C-terminal tail and its possible application in inducing the formation of hard tissues. This may also be the underlying reason why QP5 can promote the mineralization of hDPCs and the formation of tertiary dentin in direct pulp capping.

Consistent with *in vitro* results, we used a rat pulp capping model to explore the *in vivo* effect of QP5. Direct pulp capping is the most commonly used method to treat pulp exposure. Numerous studies have shown that the healing of rat molar pulp tissue after direct pulp capping is histologically comparable with that in humans. We evaluated the formation of tertiary dentin and the morphology of dental pulp using μCT and H&E staining, respectively. Corresponding with the *in vitro* results, the results of animal experiments also demonstrated the biosafety and efficacy of QP5 in the process of pulp capping. In the QP5-treated group, increased tertiary dentin formation and restricted inflammation were observed. The images of H&E staining showed almost no tertiary dentin formation occurred directly underneath the exposed area, and even extensive cell necrosis at 8 weeks in the PBS-treated group (Fig. 4Ag–i and 4Bg–i). This phenomenon indicated that the odontoblasts in this area were died due to unrestricted inflammation. The severity of tissue necrosis in the PBS-treated group was greater than that in the QP5-treated group. CH is the gold standard for indirectly accelerating the formation of tertiary dentin [33]. However, CH has been demonstrated to cause tissue necrosis at the site of direct contact, followed by internal tooth resorption, calcification, etc. [34]. These disadvantages may be responsible for the widely fluctuating success rates. In contract, a well-formed incomplete dentin bridge was observed in our samples treated with QP5 with less inflammatory response, thus we further detected the effect of QP5 on LPS-stimulated inflammatory cytokines in hDPCs *in vitro*. Unfortunately, the results showed that QP5 does not reduce the expression of IL-6 and TNF-α of hDPCs *in vitro*. Therefore, we speculated that the reduced inflammatory response *in vivo* might be attributed to the regulation effect of QP5 in establishing the mineralized barrier quickly and blocking the external stimulation, rather than the directly anti-inflammatory effect of QP5. The formation of the barrier might be due to the combination of two factors. The first factor is the promotion effect of C-terminus sequence in QP5 in inducing mineralization of hDPCs, which has been shown in *in vitro* results. Secondly, QP5 is enriched in proline, in which the carboxyl group enhances its interaction with both calcium and phosphate ions, thus the peptide could effectively promote mineral substance crystallization [35]. These properties of QP5 indicate an attempt to establish contact with the sidewalls of the dentin to block and preserve the exposed pulp, without severe inflammation seen in the dental pulp beneath the access cavity [36]. Therefore, the repair of the dental pulp was significantly superior in the QP5-group compared to the other groups.

In summary, the amelogenin-derived peptide QP5 shows the potential as a pulp therapy agent because of its significant pro-mineralization effects of hDPCs and inducing mineral deposition by adsorbing calcium and phosphorus *in vivo*, which can form a barrier to isolate external stimuli more quickly. This study also indicates that functional protein-derived peptides with a smaller size, better stability and a wide range of bioactivities, could be used as an improved alternative to large molecular proteins. However, further studies on QP5 are needed to elucidate the mechanism of action and methods of application. Suitable and reliable carriers, especially systems with delayed or controlled release, need to be identified for the clinical application of QP5 in pulp capping.

## Funding

This work was supported by the National Natural Science Foundation of China (grants number 81771062 and 81970931).

*Conflict of interest statement:* All authors declare no conflict of interest.
